# Isolation, identification, and potential probiotic characterization of isolated lactic acid bacteria and in vitro investigation of the cytotoxicity, antioxidant, and antidiabetic activities in fermented sausage

**DOI:** 10.1186/s12934-019-1239-1

**Published:** 2019-11-05

**Authors:** Nadia S. AlKalbani, Mark S. Turner, Mutamed M. Ayyash

**Affiliations:** 1 Department Food, Nutrition and Health, College of Food and Agriculture, United Arab Emirates University (UAEU), Al Ain, UAE; 2 School of Agriculture and Food Sciences, The University of Queensland (UQ), Brisbane, Australia

**Keywords:** Probiotic, Fermented sausage, Cytotoxicity, Antioxidant, Antidiabetic, Lactic acid bacteria, Fish

## Abstract

**Background:**

Probiotic bacteria can provide health benefits when delivered in functional foods. This study involved isolation of lactic acid bacteria (LAB) from traditionally dried and salted anchovy fish and characterization of their survival in simulated gastrointestinal digestion. Promising strains were used to prepare fermented fish sausages which were then evaluated for cytotoxicity activity against two cancer cell-lines, antidiabetic activity as determined by α-amylase and α-glucosidase inhibition, and antioxidant and proteolytic activities in vitro, as compared to non-fermented control sausages.

**Results:**

Out of 85 LAB obtained, 13 isolates with high tolerance to simulated gastrointestinal digestion were obtained, which were identified as *Enterococcus* spp. Four *E. faecium* strains, one *E. faecalis*, and one *E. durans* were used separately to make fermented fish sausages. The α-amylase and α-glucosidase inhibition from fish sausages fermented by *Enterococcus* spp. ranged from 29.2 to 68.7% and 23.9 to 41.4%, respectively, during 21 days of storage. The cytotoxicity activities against Caco_2_ and MCF-7 cells of fish sausages fermented with *Enterococcus* spp. ranged from 18.0 to 24% and 13.9 to 27.9%, respectively. Cytotoxicity activities correlated positively with proteolysis and antioxidant activities, α-amylase and α-glucosidase inhibition activities, but negatively with the pH in fermented fish sausages. Strains also exhibited antimicrobial activity against foodborne pathogens and presented no significant concerns with regards to antibiotic resistance or virulence gene content.

**Conclusions:**

Fish sausages fermented by potential probiotic isolates of *Enterococcus* spp. from dried fish had valuable health-promoting benefits compared with non-fermented control sausages.

## Background

Probiotics are defined as “living microorganisms which, when administrated in adequate numbers, confer a health benefit to the host” [1]. In general, commercially available probiotic bacteria are from the *Lactobacillus*, *Bifidobacterium*, *Streptococcus* and *Enterococcus* genera. The health benefits of probiotics in treating disorders, including inflammatory bowel disease, irritable bowel syndrome, constipation, antibiotic-associated and acute diarrhea, allergy-related conditions, hypertension, and diabetes, have been well-documented by numerous esteemed scientific reports and systematic reviews [2]. It is desirable for probiotic strains to possess several properties, such as a tolerance to gastrointestinal conditions (gastric, intestinal, and bile acids), attachment to epithelial cells, assimilation of cholesterol in food and the human intestine, bile salt hydrolysis, safety (no virulence genes, absence of hemolytic activity, and sensitive to antibiotics), antimicrobial properties, and survival during the fermentation process and storage [3, 4]. However, it is not essential that potential probiotics possess all of the above characteristics. The industrial characteristic such as tolerance to heat treatment, particularly spray drying, is also preferable [5]. Exopolysaccharide (EPS) production could provide health benefits to consumers as non-digestible fiber or in improving the sensory properties of food [6]. Probiotic strains may also be used to produce fermented functional foods [7]. Functional foods produced using probiotics possess superior health advantages compared with conventional food products [8]. Attempts to screen for new LAB bacteria that possess excellent probiotic characteristics from various food sources is ongoing [9–11].

In the markets of Middle Eastern and Gulf Cooperation Council (GCC) countries, the traditionally dried anchovy fish (*Engraulis encrasicolus*) have low water activity and high salt concentration (4 to 6%), which results in their long shelf-life. This food offers a potentially unique source of lactic acid bacteria (LAB) with novel probiotic properties, which may be incorporated into functional fermented foods such as fermented fish sausages. Therefore, the objectives of this study were to: (1) isolate LAB from dried anchovy fish and characterize their potential probiotic properties and (2) examine the potential health-promoting benefits (cytotoxicity activity against two cancer cell-lines, antidiabetic activity by α-amylase and α-glucosidase inhibition, and antioxidant and proteolytic activities) of fish sausages fermented by selected LAB isolates, which showed promising probiotic characteristics, in vitro.

## Results and discussion

### General characterization of LAB isolates

One hundred and fifty colonies, with different morphological properties, isolated on MRS agar from traditional fish products sold in fish markets were Gram-stained and tested for catalase. Eighty-five isolates were Gram-positive, cocci shaped, and catalase-negative; these isolates were subjected to further examination. All 85 isolates showed strong growth at 37 °C in MRS media under anaerobic conditions.

### Tolerances to gastrointestinal conditions and bile acids

In gastric conditions (pH 2.0 with pepsin), the bacterial population dropped by 0.5 to 5.2 log CFU/mL after 2 h of incubation (Additional file 1: Table S1). In intestinal conditions (pH 8.0 with trypsin), however, the reductions in the bacterial population were minor, ranging from 0.0 to 1.5 log CFU/mL after 6 h of incubation (Additional file 1: Table S1). A maximum of 2.0 log CFU/mL reduction after a combination of gastric and intestinal treatments was considered the cut-off level for tolerance. Bacterial growth suppression percentages due to oxgall, cholic, and taurocholic acids, the representative bile acids, were from 0 to 17.5%, 0 to 15.8%, and 0 to 60.6% after 3 h, and from 0 to 18%, 0 to 35.0%, and 18.1 to 63.7% after 6 h, respectively (Additional file 1: Table S2). Taurocholic acid exhibited greater inhibition compared to oxgall and cholic acids. In this study, the gastric and intestinal conditions were more influential in selecting isolates than bile. Out of the 85 isolates, 29 were selected due to their higher tolerances to gastrointestinal conditions and bile acids. All 29 isolates were identified by 16S rDNA sequencing. Out of these 29 isolates, 13 had significant tolerances and were further investigated for probiotic characterization. As shown in Additional file 1: Table S3, all 13 isolates were *Enterococcus* spp. including 4 *E. faecalis*, 7 *E. faecium* and 2 *E. durans*. In general, our tolerance results to gastrointestinal conditions are similar with those reported by El-Jeni et al. [12] and Hwanhlem et al. [13].

### Screening for virulence genes

PCR was applied to the 13 isolates to test for the presence of genes encoding the cytolysin structural subunits (*cyl*L_L_ and *cyl*L_s_), aggregation substance (*asa*1), gelatinase (*gelE*), enterococcal surface protein (*esp*), hyaluronidase (*hyl*), cell wall adhesion (*efaA*_*fs*_), aggregation protein involved in adherence to eukaryotic cells (*agg*), and adhesion of collagen protein (*ace*) (Additional file 1: Table S4). The screening results revealed that the 13 *Enterococcus* spp. tested in this study did not contain the *cyl*L_L_, *cyl*L_s_, *asa*1, *esp,* and *hyl* genes. In particular, the absence of cytolysin coding genes (*cyl*L_L_ and *cyl*L_s_) is necessary for potential food applications of *Enterococcus* strains. Generally, cytolysin is a bacterial toxin, expressed by some *Enterococcus* species, that displays both hemolytic and bactericidal activities [14]. The presence of the *efaA*_*fs*_, *agg,* and *ace* genes in the studied bacterial strains could be considered an advantage. These genes might facilitate the colonization of these strains in the human gut to provide potential health benefits, but gelatinase (*gelE*) production can contribute to the proteolytic activity of some *Enterococcus* strains, which represents a technological drawback in meat processing. However, the presence of the *gelE* gene alone does not necessarily mean that the current strains will generate gelatinase activity. Other genes belonging to gelatinase activity operon would need to be present [15].

### Antibiotic sensitivity, hemolysis, antimicrobial activity, and co-aggregation

Table 1 shows that *Enterococcus* spp. were most sensitive towards ampicillin, penicillin, and vancomycin. Lower sensitivity was observed against clindamycin, erythromycin, and trimethoprim. Antibiotic-sensitive probiotics have no contribution to the horizontal transmission of antibiotic-resistant genes between pathogens of the same species [16]. Our results are consistent with the antibiotic and antimicrobial results reported in the *Enterococcus* isolates obtained from camel milk by Ayyash et al. [17]. Fortunately, no hemolysis was detected for any of the *Enterococcus* spp strains tested (Table 1). All 13 *Enterococcus* strains exhibited antimicrobial activities against four foodborne pathogens, with the greatest inhibition being against *L. monocytogenes,* followed *S. aureus* and *S.* Typhimurium (Table 1). The potential capabilities of the *Enterococcus* spp. isolates to displace pathogens in the gastrointestinal tract is presented by co-aggregation analyses (Additional file 1: Table S5). *Enterococcus* spp. isolates here showed the greatest co-aggregation with *S.* Typhimurium compared to the 3 other pathogens (Additional file 1: Table S5).

**Table 1:** Antimicrobial activity against four pathogens and antibiotic resistance towards six different antibiotics

Bacteria	Antimicrobial activity^a^				Antibiotic resistance^b^						Hemolysis
	*E. coli* O157:H7	*S.* Typhimurium	*L. monocytogenes*	*S. aureus*	PEN	TRI	AMP	CLI	VAN	ERY	
*E. faecalis* MF067467	+	+	++	++	MS	MS	S	S	S	S	−
*E. faecalis* MF067469	+	+	+	++	MS	S	MS	R	MS	MS	−
*E. faecium* MF067470	+	++	+++	++	MS	S	S	S	S	MS	−
*E. faecium* MF067487	+	++	+++	++	S	MS	S	R	S	MS	−
*E. faecium* MF067495	+	++	+++	++	S	R	S	S	S	MS	−
*E. faecalis* MF067500	+	+	++	++	S	R	S	R	S	MS	−
*E. faecium* MF067509	+	+	++	+	S	R	S	MS	S	R	−
*E. faecium* KY962871	++	++	+++	++	S	MS	S	S	S	MS	−
*E. faecium* KY962874	++	+	+	+	S	MS	S	S	R	S	−
*E. durans* KY962882	+	+	++	+	S	R	S	S	S	S	−
*E. faecium* KY962883	+	+	++	++	S	MS	S	MS	S	MS	−
*E. durans* KY962888	+	+	+	+	S	R	S	R	S	S	−
*E. faecalis* KY962905	+	+	+	++	S	R	S	R	S	MS	−

*R* resistant, *MS* moderately susceptible, *S* susceptible. The interpretive zones for each antibiotic were assigned according to Charteris et al. [31]

^a^(−) no inhibition; (+) inhibition zone 0.1 to 1.0 mm; (++) inhibition zone 1.1 to 2.0 mm; (+++) inhibition zone > 2.1 mm

^b^PEN: penicillin (10 µg); TRI: trimethoprim (25 µg); AMP: ampicillin (10 µg); CLI: clindamycin (2 µg); VAN: vancomycin (30 µg); ERY: erythromycin (15 µg)

### Probiotic characterization

#### Autoaggregation, hydrophobicity, cholesterol removal, bile salt hydrolysis (BSH), and conjugated linoleic acid (CLA) conversion

Table 2 shows the autoaggregation (%), hydrophobicity (%), BSH (Unit/mg), cholesterol removal (%), and CLA conversion (%) of the selected 13 *Enterococcus* spp. After 3 h and 24 h autoaggregation ranged from 8.2 to 21.3% and 29.0 to 67.0%, respectively. The hydrophobicity results were relatively low, except for *E. faecalis* MF067467 and MF067469, which had hydrophobicity of > 44.0% and > 27.0%, respectively (Table 2). Autoaggregation and hydrophobicity are indicative parameters for cell surface properties of probiotics, which indicate a potential higher adherence to epithelial cells [18, 19]. Our results indicate a higher adherence ability of than those reported by Ayyash et al. [17] and Das et al. [5].

**Table 2:** Autoaggregation, hydrophobicity, bile salt hydrolysis (BSH), cholesterol removal, conjugated-linoleic acid (CLA) conversion of 13 *Enterococcus* spp

Bacteria	Auto-aggregation (%)		Hydrophobicity (%)			BSH (U/mg)	Cholesterol removal %	CLA %
	3 h	24 h	Hexadecane	Xylene	Octane			
*E. faecalis* MF067467	20.8 ± 0.22^ab,1^	67.4 ± 0.00^a^	44.4 ± 1.8^a^	44.1 ± 2.6^a^	46.2 ± 3.7^a^	3.9 ± 0.05^bc^	38.5 ± 2.6^f^	1.69 ± 0.25^e^
*E. faecalis* MF067469	21.3 ± 0.10^c^	64.9 ± 0.02^c^	34.5 ± 3.5^b^	27.1 ± 2.7^b^	40.1 ± 1.2^a^	5.3 ± 0.71^bc^	6.5 ± 1.7^g^	1.21 ± 0.18^ef^
*E. faecium* MF067470	11.4 ± 0.68^c^	48.0 ± 0.44^c^	3.9 ± 0.4^efg^	0.3 ± 0.0^g^	1.7 ± 0.2^ef^	4.2 ± 0.49^b^	54.1 ± 4.6^ab^	2.11 ± 0.32^de^
*E. faecium* MF067487	8.2 ± 0.68b^c^	34.1 ± 0.27^abc^	0.8 ± 0.1^fg^	16.0 ± 1.6^cd^	4.4 ± 0.4^ef^	5.2 ± 0.72^def^	40.9 ± 6.7^ef^	1.69 ± 0.25^e^
*E. faecium* MF067495	13.9 ± 0.57^a^	46.0 ± 0.48^ab^	13.6 ± 1.4^cd^	13.1 ± 1.3^cde^	14.2 ± 1.4^cd^	4.7 ± 0.25^a^	51.4 ± 4.8^bc^	1.2 ± 0.18^ef^
*E. faecalis* MF067500	9.1 ± 0.74^abc^	29.1 ± 0.63^abc^	1.2 ± 0.1^fg^	4.2 ± 0.4^efg^	3.5 ± 0.4^ef^	4.1 ± 0.33^gh^	39.4 ± 1.0^f^	1.24 ± 0.19^ef^
*E. faecium* MF067509	12.2 ± 0.71^abc^	36.4 ± 1.75^bc^	5.7 ± 0.6^defg^	16.0 ± 1.6^cd^	7.6 ± 0.8^def^	3.9 ± 0.11^cde^	53.6 ± 1.2^ab^	0.95 ± 0.14^f^
*E. faecium* KY962871	11.6 ± 0.63^abc^	42.5 ± 0.08^abc^	1.4 ± 0.1^efg^	0.5 ± 0.0^fg^	1.7 ± 0.2^ef^	3.0 ± 0.04^efg^	46.6 ± 1.4^cde^	0.7 ± 0.10^f^
*E. faecium* KY962874	8.2 ± 0.61^ab^	35.3 ± 0.30^abc^	3.9 ± 0.4^defg^	3.4 ± 0.3^def^	2.7 ± 0.3^de^	4.5 ± 0.25^b^	48.8 ± 3.1^bcd^	43.2 ± 6.48^a^
*E. durans* KY962882	8.2 ± 0.48^abc^	37.4 ± 0.43^c^	9.2 ± 0.9^def^	9.3 ± 0.9^def^	21.8 ± 2.2^b^	5.7 ± 0.95^gh^	59.1 ± 1.0^a^	21.8 ± 3.26^b^
*E. faecium* KY962883	10.6 ± 0.89^abc^	34.7 ± 0.57^abc^	0.8 ± 0.1^g^	1.3 ± 0.1^fg^	0.6 ± 0.1^f^	5.1 ± 0.60^bc^	51.9 ± 5.3^bc^	2.5 ± 0.38^de^
*E. durans* KY962888	14.9 ± 0.59^abc^	40.8 ± 0.82^c^	9.4 ± 0.9^de^	18.4 ± 1.8^c^	13.1 ± 1.3 ^cd^	6.0 ± 0.31^h^	40.4 ± 1.0^f^	6.39 ± 0.96^c^
*E. faecalis* KY962905	18.1 ± 0.26^abc^	57.1 ± 0.48^abc^	19.8 ± 2.0^c^	14.4 ± 1.4^cd^	7.8 ± 0.8^bc^	4.0 ± 0.75^cde^	43.0 ± 3.6^def^	7.15 ± 1.07^c^

^a–g^Means in the same column with different lowercase letters differed significantly (*p* < 0.05)

^1^Values are mean ± standard error of triplicates

Excluding *E. faecalis* MF067469, the 13 *Enterococcus* spp. exhibited marked cholesterol removal capabilities, ranging from 38.0 to 59.0%. Moreover, BSH activities were notable and varied from 3.0 to 6.0 U/mg (Table 1). Several mechanisms have been postulated for cholesterol assimilation by probiotic bacteria, including cholesterol incorporation in the cell envelope, conversion of cholesterol to coprostanol by a reductase, and disruption of cholesterol micelles in the intestine by BSH [20]. Both the cholesterol and BSH results are superior to those reported by Ayyash et al. [17]. Table 1 exhibits that *E. faecium* KY962874 and *E. durans* KY962882 had pronounced CLA conversions 43.2% and 21.8%, respectively. CLA has a significant impact on human health [21].

#### Lysozyme and heat resistance, and EPS production

All *Enterococcus* spp. had excellent industrial characteristics (Additional file 1: Table S6). The bacterial population decreased slightly (*p* > 0.05) as a result of lysozyme and heating treatments, except *for E. faecalis* MF067467. The 13 *Enterococcus* spp. performed better than those reported by Teles et al. [22], who isolated LAB from cocoa fermentation. All strains displayed the potential to produce EPS, except *E. faecium* MF067487 (Additional file 1: Table S6).

### Fermented fish sausage

Based on previous characteristics (especially cholesterol removal, CLA, antibiotic resistance, antimicrobial production, autoaggregation, and hydrophobicity), the following six strains were selected to prepare functional fermented fish sausages: *E. faecium* MF047470, MF047495, MF047509, and KY962874, *E. faecalis* KY962905, and *E. durans* KY962882.

#### LAB population, pH, and TBAR

The 6 *Enterococcus* spp. maintained high bacterial populations > 7.0 log CFU/g during 21 days of storage (Additional file 1: Table S7). pH values declined (*p* < 0.05) after 24 h of fermentation at 37 °C to a pH of approximately 4.6, then remained constant. The pH reduction in fish sausages fermented by *Enterococcus* spp. was faster compared than that of the samples inoculated with the starter culture only (commercial) (Additional file 1: Table S7). Our results harmonize with the FAO/WHO [1] guidelines, which recommend probiotics to be administrated in high viable numbers. The pH values in control sausages did not fall during storage. Lipid oxidation measured by TBAR values was relatively low at < 0.7 mg MDA/kg for all samples at each time point, but the values increased slightly during the storage period in some samples (Additional file 1: Table S7).

#### Degree of protein hydrolysis (DH%)

**Fig. 1:**
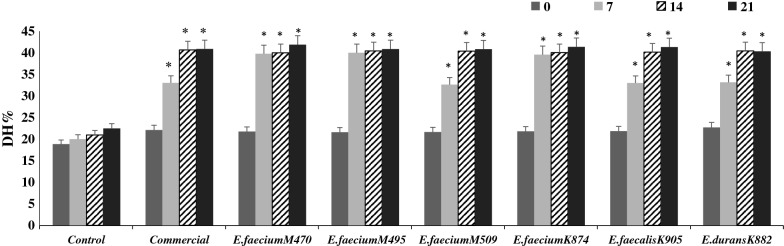
Degree of hydrolysis (%) of non-fermented (control) and fermented fish sausages during 21 days of storage. *means had a significant difference at p < 0.05 compared with the control at the equivalent day

DH% increased (*p* < 0.05) from 20 to around 40% during 21 days of storage (Fig. 1). The 6 *Enterococcus* spp. had comparable proteolytic activity compared with the commercial starter culture. An ANOVA showed no significant differences in DH% between fermented fish sausages after 21 days. As shown in Fig. 1, only control fish sausage (without inoculated bacteria) had lower DH% (*p* < 0.05) than fermented sausages. Fish sausage fermented with *E. faecium* MF047470, MF047495, and KY962874 had a better DH% during 7 days of storage than the remaining isolates (Fig. 1).

Proteolysis plays a major role in sausage fermentation. The intact meat proteins are degraded into peptides and free amino acids which have a significant impact on the health-promoting benefits and physicochemical properties of fermented sausages [23]. The proteolytic activity in fish sausages may be attributed to: (1) the endogenous fish protease systems, especially cathepsins which are acidic enzymes [24]; pH values of approximately 4.0 may activate cathapsins in fermented sausage; (2) proteolytic enzymes produced by *Enterococcus* spp. or the commercial culture. To the best of our knowledge, there is a lack of information related to proteolysis that occurs in fermented fish sausage. A Pearson’s test showed that DH% had significant positive (r = 0.881) and negative (r = − 0.771) correlations with the bacterial isolates and pH values, respectively (Additional file 1: Table S8).

#### Antioxidant activities

**Fig. 2:**
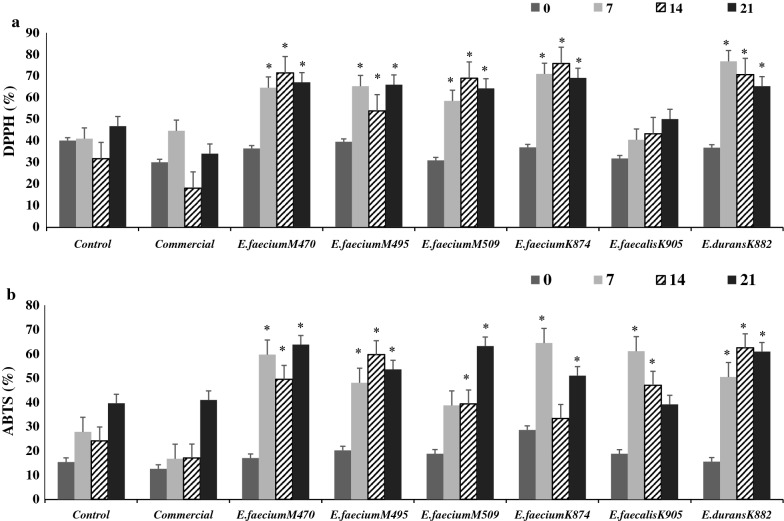
Antioxidant activities measured by DPPH % (**a**) and ABTS% (**b**) of non-fermented (control) and fermented fish sausages during 21 days of storage. *Means had a significant difference at p < 0.05 compared with the control at the equivalent day

DPPH% ranged from 30 to 64% in fish sausages fermented with *Enterococcus* spp. isolates (Fig. 2a), whereas the ABTS% ranged from 19.8 to 55.3% (Fig. 2b). Fish sausages fermented by *Enterococcus* spp. had greater DPPH% compared with the commercial culture and control sausages (Fig. 2a). DPPH% and ABTS% also increased (*p* < 0.05) during 7 days of storage (Fig. 2a, b).

Several biochemical changes are taking place in the fermented sausages resulting in bioactive compound generation. The bioactive compounds have a significant role in mitigating the influence of free radical reactive oxygen species, such as superoxide (·O2‾, ·OOH), hydroxyl (·OH), and peroxyl (ROO·) radicals [25]. Typically, the free radical is neutralized via electron donation by the bioactive compound [26]. The significant and positive correlations between DPPH% and DH% (r = 0.329), and ABTS% and DH% (r = 0.312) might explain the antioxidant results in fermented fish sausage (Additional file 1: Table S8). The increase in DPPH% and ABTS% during storage suggests that peptides released as a result of proteolysis may possess antioxidant activities.

#### Antidiabetic activity by α-amylase and α-glucosidase inhibition

**Fig. 3:**
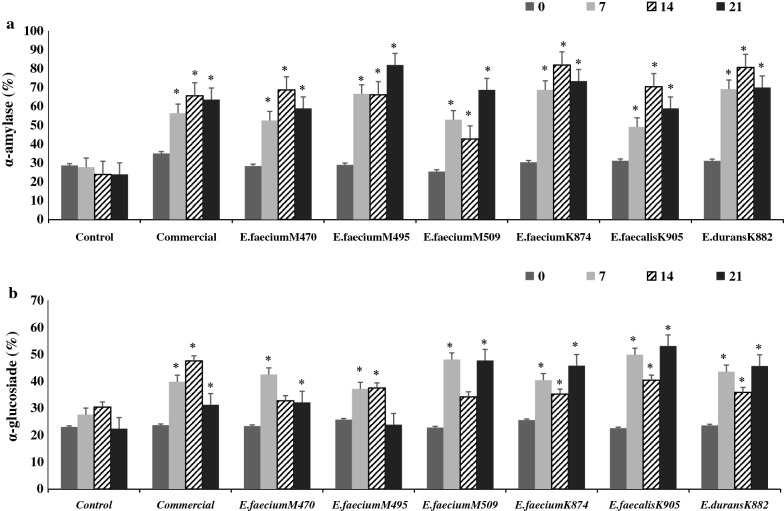
Inhibition of amylase (**a**) and glucosidase (**b**) by non-fermented (control) and fermented fish sausages during 21 days of storage. *Means had a significant difference at p < 0.05 compared with the control at the equivalent day

Figure 3a, b illustrate the increase in α-amylase and α-glucosidase inhibition by fermented fish sausages during storage which was greater (*p* < 0.05) than for the control. The amylase and glucosidase inhibition in fish sausages fermented by *Enterococcus* spp. extended from 29.2 to 68.7% and 23.9 to 41.4%, respectively, during 21 days of storage. In general, fish sausage fermented by *E. faecalis* KY962905 had greater (p < 0.05) α-glucosidase inhibition followed by *E. faecium* MF067509 compared with other strains (Fig. 3b). The stains *E. faecium* MF067495, *E. faecium* KY962874 and *E. durans* KY962882 exhibited higher (p < 0.05) inhibitions of α-amylase activities compared with other strains (Fig. 3a). These differences between strains could be attributed to the variation in proteolytic activities between strains.

A practical approach to managing diabetes via diminishing carbohydrate hydrolysis is to inhibit α-amylase and α-glucosidase activities [27]. The released peptides due to proteolysis during sausage fermentation may be accountable for the inhibition of α-amylase and α-glucosidase enzymes [28]. Amylase and glucosidase inhibition correlated positively with bacterial population, DH%, DPPH%, and ABTS%, and negatively with pH (Additional file 1: Table S8). To the best of our knowledge, no information is available about antidiabetic activities of fermented fish sausages. The current α-amylase and α-glucosidase inhibition in fish sausage fermented by the 6 *Enterococcus* spp. were found to be greater than those in bovine sausages fermented by *L. plantarum* [8], however if these LAB activities have biological significance in the human body remains to be determined.

#### Cytotoxicity activity

**Fig. 4:**
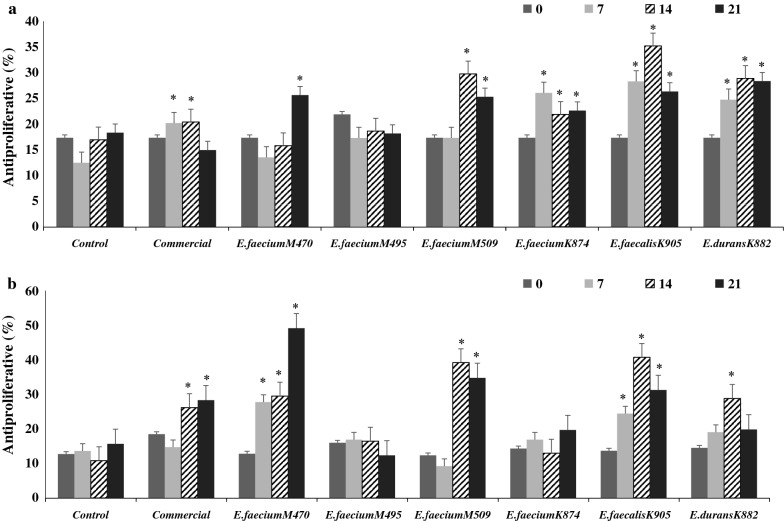
Cytotoxicity activities against Caco_2_ (**a**) and MCF-7 (**b**) of non-fermented (control) and fermented fish sausages during 21 days of storage. *Means had a significant difference at p < 0.05 compared with the control at the equivalent day

The cytotoxicity activities against the colon-cancer cell-line, Caco_2_, and the breast cancer cell-line, MCF-7, by fish sausages fermented with *Enterococcus* spp. ranged from 18.0 to 24% (Fig. 4a) and 13.9 to 27.9% (Fig. 4b), respectively. As shown in Fig. 4a, the cytotoxicity activities of fish sausages fermented by *E. faecium* MF067509, *E. faecium* KY962874, *E. faecalis* KY962905, and *E. durans* KY962882 against Caco_2_ cells were significantly different compared with the control and commercial culture fermented sausages. In addition, fish sausages fermented by *E. faecium* MF067470, *E. faecium* MF067509, and *E. faecalis* KY962905 had pronounced cytotoxicity activity against MCF-7 cells (Fig. 4b).

There are two primary mechanisms of cytotoxicity activity against tumors: (1) the peptides released as a result of proteolysis compete with cancer growth factors for cancer cell-membrane receptors; (2) the released peptides may induce apoptosis in cancer cells [29]. The intermediate and small peptides released during fish sausage fermentation may explain the cytotoxicity activities. The nature of these peptides might play a role, because not all fermented fish sausages showed significant cytotoxicity activity compared with the control. Further work identifying potential bioactive peptides would be of interest. Cytotoxicity activities correlated positively with DH%, DPPH%, ABTS%, amylase, and glucosidase inhibition, and negatively with pH (Additional file 1: Table S8).

## Conclusions

Dried fish products were identified as novel sources of LAB with desirable probiotic characteristics. *Enterococcus* spp. were able to tolerate gastric and intestinal conditions, lower cholesterol, produce CLA, and hydrolyze bile salts. The safety assessment of these isolates with regards to antibiotic resistance and virulence properties did not present any concerns. The fermented fish sausages containing *Enterococcus* spp. exhibited noticeable cytotoxicity and antioxidant features. The *E. faecium* MF067509, *E. faecalis* KY962905, and *E. durans* KY962882 have promising probiotic features, and have the potential to be used by the meat processing industry to develop new functional fermented foods.

## Methods

### Sample collection

One-hundred and fifty samples of traditional dried fish were collected from different fish markets in UAE (Abu Dhabi, Dubai, Sharjah, and Al-Ain). Samples were transported in an ice-box and directly tested in our food microbiology lab at UAE University upon arrival. All chemicals in this study were purchased from Sigma-Aldrich (St. Louis, MO, USA) unless stated otherwise.

### Isolation of lactic acid bacteria (LAB)

LAB were isolated on MRS agar (LAB-M, Bury, UK) after an incubation with MRS broth supplemented with 2% NaCl for 12 h at 37 °C. Plates were incubated at 37 °C for 24 h aerobically. The Gram-staining and catalase tests were performed for 150 colonies with different morphological properties. Only Gram-positive and catalase-negative colonies were sub-cultured in MRS broth (LAB-M). Glycerol stocks (50% v/v) were prepared for each colony and stored at − 80 °C.

### Tolerance to stimulated gastrointestinal conditions and bile salts

The tolerance to gastric (pH 2.0 + pepsin for 2 h) and intestinal (pH 8.0 + trypsin for 6 h) conditions were performed according to methods detailed by Saelim et al. [30]. A reduction of < 2.0 logs was considered as tolerant to gastrointestinal conditions. The bile salt tolerance was carried out according to Ayyash et al. [17]. The growth suppression (%) by 1.0% oxgall, 0.3% cholic, and 1.0% taurocholic acids was calculated using the following Eq. (1):

1 $$ \%  \;of\;growth\;suppression = \frac{{OD_{600}  \;Control\;broth {-}OD_{600}  \;bile\;broth }}{{OD_{600} \;control\;broth}} \times 100. $$

The tolerance results of the isolates in the simulated gastrointestinal conditions and bile conditions are presented in supplementary data Additional file 1: Tables S1 and S2, respectively.

### Identification of selected isolates by 16S rDNA sequencing

LAB identification was carried out using 16S rDNA sequence analysis of selected strains which was amplified by PCR procedure described by Ayyash et al. [17]. PCR primers 27F (5^′^-AGAGTTTGATCCTGGCTCAG-3^**′**^) and 1492R (5^**′**^**-**TACGGYTACCTTGTTACGACTT-3^**′**^) were used for amplification. Isolate names and GenBank accession numbers are presented in Additional file 1: Table S3.

### Safety assessment

#### Screening for virulence genes

The selected LAB isolates were screened for the presence of nine virulence genes using the method described by Hwanhlem et al. [13]. Virulence genes results are presented in Additional file 1: Table S4. *E. faecalis* DSM 20478 was used as a positive control for PCR.

#### Antibiotic susceptibility

Antibiotic-resistant was measured according to the method described by Das et al. [5]. Penicillin (PEN; 10 µg), trimethoprim (TRI; 25 µg), ampicillin (AMP; 10 µg), clindamycin (CLI; 2 µg), vancomycin (VAN; 30 µg), and erythromycin (ERY; 15 µg) were the antibiotics tested. Antibiotic disks and cartridge dispensers were from Oxoid (Thermo Fisher Scientific, Dardilly, France). The interpretative zones to resistant (R), moderately susceptible (MS) and susceptible (S) were assigned according to Charteris et al. [31].

#### Hemolytic activity

Hemolytic activity of LAB isolates was examined on Colombia blood agar (Himedia, Mumbai, India) according to Angmo et al. [32].

### Antimicrobial activities

#### Antibacterial activity

Antibacterial activity of cell-free supernatant of LAB isolates against *Listeria monocytogenes* ATCC 7644, *Salmonella* Typhimurium 02-8423, *Escherichia coli* O157:H7 1934, and *Staphylococcus aureus* ATCC 15923 was tested according to Mishra and Prasad [33] using the disc diffusion method. These foodborne pathogens were acquired from Prof. Richard Holley’s Laboratory, University of Manitoba, Canada. These pathogens were chosen due to their association with foodborne outbreaks [34].

#### Coaggregation

Coaggregation of LAB isolates and four pathogens was assayed at 37 °C during incubation for 4 h according to method detailed in [35]. The coaggregation percentage was expressed as Eq. (2). Results are presented in Additional file 1: Table S5.

2 $$ {\text{Coaggregation \% }} = \frac{{A_{0} - A_{t} }}{{A_{0} }} \times 100. $$

where A_t_ represent absorbance at time t and A_0_ represent absorbance at t = 0.

### Evaluation of probiotic characteristics

#### Autoaggregation

Autoaggregation was measured according to the method described by Collado et al. [36]. The autoaggregation percentage was calculated based on Eq. (3):

3 $$ {\text{Autoagreggation \%  }} = \left[ {1 - \frac{{A_{t} }}{{A_{0} }}} \right] \times 100, $$

where A_t_ represent absorbance at time t and A_0_ represent absorbance at t = 0.

#### Hydrophobicity

Hydrophobicity was tested against three hydrocarbons (n-hexadecane, xylene, and octane). The hydrophobicity assay and calculations (%) were carried out according to a previously published method [33].

#### Bile salt hydrolysis (BSH)

BSH activities of pure isolates were measured by determining the amount of amino acids released from conjugated bile salts by LAB strains according to the method described by Liong and Shah [37]. BSH activities were assayed against bile salt mixture (6 mM; glycocholic acid, glycochenodeoxycholic acid, taurocholic acid, taurochenodeoxycholic acid, and taurodeoxycholic acid).

#### Cholesterol removal

Cholesterol removal by LAB was determined according to the method described in Ayyash et al. [17] without modifications.

#### Conjugated linoleic acid (CLA) conversion

CLA conversation capabilities were screened using a UV-spectrometer method as described by Vieira et al. [21] with minor modifications. Sterilized MRS broth was mixed with free linoleic acid (LA; 1 mg/mL) and 2% Tween 80. The MRS-LA was inoculated with 1% of activated culture and incubated for 48 h at 30 °C, 35 °C, and 40 °C, individually. A standard curve (20–160 µg/mL) was prepared from the reference t10, c12 CLA isomer to quantify total CLA. The CLA conversion was calculated by Eq. (4):

4 $$ {\text{\%  CLA conversion}} = \frac{{{\text{Abs at }}48\;{\text{h}} - {\text{Abs at }}0   {\text{h}}}}{{{\text{Abs at }}0   {\text{h}}}} \times 100. $$

#### Lysozyme tolerance, heat resistance, and exopolysaccharides (EPS) production

LAB isolate tolerance toward lysozyme during 90 min of incubation at 37 °C was determined according to the method of Vizoso Pinto et al. [38]. Heat resistance at 60 °C for 5 min for selected bacterial isolates was carried out according to the method found in Teles et al. [22].The ability of the LAB isolates to produce EPS (positive/negative) was measured according to the method described in Angmo et al. [32].

### Fermented fish sausage

#### Culture propagation for fish sausage

For culture growth, a 100 μL aliquot of each strain was individually transferred into MRS broth (9.9 mL) and incubated at 37 °C for 24 h. For each strain, two successive culture transfers were carried out in MRS broth prior to the experimental day. The commercial starter culture for sausage fermentation consisted of *Pediococcus pentosaceus* and *Staphylococcus carnosus* (positive control), and was kindly provided by Chr-Hansen Holding A/S (Horsholm, Denmark).

#### Fish sausage making

Frozen fish fillets were purchased from local market in Al-Ain, UAE. The fermented fish sausages were prepared according to Sachindra and Mahendrakar [39] with some formula modifications. The formulation of fish sausage included 560 g of fish meat, 14.3 g of salt, 10.7 g of sugar, 1.4 g of sodium tripolyphosphate, 0.8 g of pepper powder, 0.8 g of garlic powder, 65 g of cornstarch, 35 mL of refined vegetable oil, and 70 mL of chilled water. The sausage mixture (700 g) was prepared by mixing the ingredients in sequence in a bowl chopper and inoculating it with active culture (10^7^–10^8^ CFU). The inoculated mixture was stuffed into collagen casings with a 3 cm diameter. Sausage batters were vacuum-packaged (Day 0) and fermented at 37 °C until the pH was < 5.0 (approximately 24 h). Afterwards, vacuumed-packaged fish sausages were stored at 4 °C for 21 days. Sausages inoculated with commercial starter culture were considered as the positive controls (commercial), whereas non-inoculated sausages were considered as the negative control (control). Sausages were sampled at 0, 7, 14, and 21 days. Day 0 represents the sausage after it was vacuumed-packaging but before fermentation.

#### LAB enumeration, pH, and lipid peroxidation by TBAR test

The LAB population in fermented sausages was enumerated according to Mejri et al. [40]. LAB populations were enumerated on MRS agar (LAB-M), and the plates (duplicate) were incubated anaerobically at 37 °C for 48 h using an anaerobic jar system (Don Whitley Scientific Limited, West Yorkshire, UK). pH values of fermented sausages were measured after mixing one portion of sausage with two portions of distilled deionized (dd)-water using a digital pH meter. Thiobarbituric acid-reactive substances (TBAR) were determined spectrophotometrically as described by Berardo et al. [41]. The TBARS value was expressed as mg malonaldehyde/kg (mg MDA/kg) of sample.

#### Water-soluble extract (WSE)

WSE was prepared by homogenizing 15 g of the fermented sausage with 60 mL of dd-water at 20,000 rpm for 30 s. After filtration through No. 1 Whatman^®^ filter paper, a clear supernatant was stored at − 20 °C for further analysis [42]. Ahead of each analysis, stored WSEs were vortexed for 1 min followed by centrifugation at 10,000×*g* for 5 min.

#### Degree of hydrolysis (DH%)

The DH% was determined using the OPA method as described by Sah et al. [43]. Proteolysis results are presented as absorbance at 340 nm. Degree of hydrolysis was determined using Eq. (5).

5 $$ {\text{DH }}\left( {\text{\% }} \right) = \frac{\text{h}}{{h_{tot} }} \times 100, $$

where h_tot_ was the total number of peptide bonds per protein equivalent; for meat, the h_tot_ value was 7.6 mEq/g protein [44], and h was the number of hydrolyzed bonds, which was determined by Eq. (6).

6 $$ h = \frac{Serine \ NH2 - \beta }{\alpha }. $$

For meat, ⍺ = 1.0 and β = 0.40 mEq/g protein [44], and the value of Serine-NH_2_ was determined using Eq. (7):

7 $$ Serine{\text{-}}NH2_{{{\raise0.7ex\hbox{${mEq}$} \!\mathord{\left/ {\vphantom {{mEq} {g \,protein}}}\right.\kern-0pt} \!\lower0.7ex\hbox{${g protein}$}}}} = \frac{{\left( {A_{sample} - A_{blank} } \right)}}{{\left( {A_{standard} - A_{blank} } \right)}} \times Con. \  of \  Serine \;Std \left( {\frac{mEq}{L}} \right) \times V \times \frac{100}{X} \times P, $$

where, V = final volume make-up of the sample in liters; X = weight of meat sample in grams; P = protein % (w/w) in sausage sample.

#### Antidiabetic activities

##### α-Amylase inhibition assay

The ⍺-amylase inhibition assay of the WSEs was carried out according to the method described by Ayyash et al. [27].

##### α-Glucosidase inhibition assay

⍺-Glucosidase inhibition assay of the WSEs was carried out according to the method in [45] with some modifications detailed in [27]. The inhibition percentage was calculated by Eq. (8):

8 $$ \upalpha{\text{-Glucosidase inhibition}} \% = \left( {1 - \frac{{Abs _{sample} - Abs _{blank} }}{{Abs_{ control} }}} \right) \times 100. $$

#### Antioxidant activity

##### Radical scavenging rate by DPPH assay

The determination of radical scavenging activity of the WSEs by the 1,1-diphenyl-2-picrylhydrazyl (DPPH) assay was performed according to Elfahri et al. [46]. The percentage of radical scavenging activity was expressed as scavenging rate % as Eq. (9):

9 $$ {\text{Scavenging rate}} \% = \left( {1 - \frac{{Abs _{sample} }}{{Abs _{blank} }}} \right) \times 100. $$

##### Radical scavenging rate by ABTS assay

The radical scavenging rate of the WSEs by the 2,2′-azino-bis(3-ethylbenzo-thiazoline-6-sulphonic acid) (ABTS^•+^) method was determined according to the procedure in Ayyash et al. [27]. Radical scavenging activity was calculated with Eq. (10):

10 $$ {\text{Scavenging rate }}\% = \left( {\frac{{Abs _{blank} - Abs _{sample} }}{{Abs _{blank} }}} \right) \times 100. $$

#### Cytotoxicity activities

The WSEs were assayed against Caco_2_ and MCF-7 carcinoma cell lines according to the method detailed by Ayyash et al. [27]. The cytotoxicity percentage was calculated by Eq. (11):

11 $$ {\text{Cytotoxicity }}\left( {\text{\% }} \right) = \left[ {1 - \frac{{{\text{R}}_{sample} - R_{o} }}{{{\text{R}}_{ctrl} - R_{o} }}} \right] \times 100, $$

where R_sample_ is the absorbance ratio of OD_570_/OD_605_ in the presence of the WSE. R_ctrl_ is the absorbance ratio of OD_570_/OD_605_ in the absence of the WSE (vehicle control). R_o_ is the averaged background (non-cell control) absorbance ratio of OD_570_/OD_605_.

### Statistical analysis

For probiotic characterization, a one-way ANOVA test was carried out to examine the significant differences in LAB isolates on the quantitative parameters (*p* < 0.05). A Fisher’s test was employed to examine differences between means at *p* < 0.05. All tests were repeated at least three times to calculate the means and standard error unless otherwise mentioned. The trials for the fermented fish sausages were conducted in triplicate on three different occasions. Each sample was assayed in duplicate unless otherwise mentioned. A one-way ANOVA was carried out to investigate the effect of the probiotic strains, at the same storage period, on the fermented sausage’s parameters (*p* < 0.05). Mean comparisons were performed using Fisher’s test (*p* < 0.05) on the same probiotic strain or storage time. A Pearson’s test was carried out to find any correlations between the health-promoting parameters for the fermented fish sausages. Correlation coefficients are presented in supplementary data (Additional file 1: Table S8). All statistical analyses were carried out using Minitab 17.0 software (Minitab Inc., PA, USA).

## Supplementary information

**Additional file 1.** Additional tables.

## Data Availability

The datasets used and/or analyzed during the current study are available from the corresponding author on reasonable request.

## References

[1] FAO/WHO: FAO/WHO working group report on drafting guidelines for the evaluation of probiotics in food. London, Ontario, Canada, April 30 and May 1, World Health Organization, http://www.who.int/foodsafety/fs_management/en/probiotic_guidelines.pdf. 2002.

[2] Hill C, Guarner F, Reid G, Gibson GR, Merenstein DJ, Pot B, Morelli L, Canani RB, Flint HJ, Salminen S (2014). Expert consensus document: The International Scientific Association for Probiotics and Prebiotics consensus statement on the scope and appropriate use of the term probiotic. Nat Rev Gastroenterol Hepatol.

[3] Khan SU (2014). Probiotics in dairy foods: a review. Nutr Food Sci.

[4] Naidu A, Bidlack W, Clemens R (1999). Probiotic spectra of lactic acid bacteria (LAB). Crit Rev Food Sci Nutr.

[5] Das P, Khowala S, Biswas S (2016). In vitro probiotic characterization of *Lactobacillus casei* isolated from marine samples. LWT Food Sci Technol.

[6] Rahbar Saadat Y, Yari Khosroushahi A, Pourghassem Gargari B (2019). A comprehensive review of anticancer, immunomodulatory and health beneficial effects of the lactic acid bacteria exopolysaccharides. Carbohydr Polym.

[7] Ayyash M, Johnson SK, Liu S-Q, Mesmari N, Dahmani S, Al Dhaheri AS, Kizhakkayil J (2019). In vitro investigation of bioactivities of solid-state fermented lupin, quinoa and wheat using *Lactobacillus* spp. Food Chem.

[8] Ayyash M, Liu S-Q, Al Mheiri A, Aldhaheri M, Raeisi B, Al-Nabulsi A, Osaili T, Olaimat A (2019). In vitro investigation of health-promoting benefits of fermented camel sausage by novel probiotic *Lactobacillus plantarum*: a comparative study with beef sausages. LWT.

[9] Abushelaibi A, Al-Mahadin S, El-Tarabily K, Shah NP, Ayyash M (2017). Characterization of potential probiotic lactic acid bacteria isolated from camel milk. LWT-Food Sci Technol.

[10] Maleki Kakelar H, Barzegari A, Hanifian S, Barar J, Omidi Y (2019). Isolation and molecular identification of Lactobacillus with probiotic potential from abomasums driven rennet. Food Chem.

[11] Plessas S, Nouska C, Karapetsas A, Kazakos S, Alexopoulos A, Mantzourani I, Chondrou P, Fournomiti M, Galanis A, Bezirtzoglou E (2017). Isolation, characterization and evaluation of the probiotic potential of a novel Lactobacillus strain isolated from Feta-type cheese. Food Chem.

[12] El-Jeni R, El Bour M, Calo-Mata P, Böhme K, Fernández-No IC, Barros-Velázquez J, Bouhaouala-Zahar B (2016). In vitro probiotic profiling of novel *Enterococcus faecium* and *Leuconostoc mesenteroides* from Tunisian freshwater fishes. Can J Microbiol.

[13] Hwanhlem N, Ivanova T, Biscola V, Choiset Y, Haertlé T (2017). Bacteriocin producing *Enterococcus faecalis* isolated from chicken gastrointestinal tract originating from Phitsanulok, Thailand: isolation, screening, safety evaluation and probiotic properties. Food Control.

[14] H-Kittikun A, Biscola V, El-Ghaish S, Jaffrès E, Dousset X, Pillot G, Haertlé T, Chobert J-M, Hwanhlem N (2015). Bacteriocin-producing *Enterococcus faecalis* KT2W2G isolated from mangrove forests in southern Thailand: purification, characterization and safety evaluation. Food Control..

[15] Ahmadova A, Dimov S, Ivanova I, Choiset Y, Chobert J-M, Kuliev A, Haertlé T (2011). Proteolytic activities and safety of use of Enterococci strains isolated from traditional Azerbaijani dairy products. Eur Food Res Technol.

[16] McEntire JC, Montville TJ, Doyle MP, Buchanan RL (2007). Antimicrobial resistance. Food microbiology: fundamentals and frontiers.

[17] Ayyash M, Abushelaibi A, Al-Mahadin S, Enan M, El-Tarabily K, Shah N (2018). In-vitro investigation into probiotic characterisation of *Streptococcus* and *Enterococcus* isolated from camel milk. LWT Food Sci Technol.

[18] Botes M, Loos B, van Reenen CA, Dicks LM (2008). Adhesion of the probiotic strains *Enterococcus mundtii* ST4SA and *Lactobacillus plantarum* 423 to Caco_2_ cells under conditions simulating the intestinal tract, and in the presence of antibiotics and anti-inflammatory medicaments. Arch Microbiol.

[19] Duary RK, Rajput YS, Batish VK, Grover S (2011). Assessing the adhesion of putative indigenous probiotic lactobacilli to human colonic epithelial cells. Indian J Med Res.

[20] Choi SB, Lew LC, Yeo SK, Parvathy SN, Liong MT (2015). Probiotics and the BSH-related cholesterol lowering mechanism: a Jekyll and Hyde scenario. Crit Rev Biotechnol.

[21] Vieira CP, Cabral CC, da Costa Lima BR, Paschoalin VMF, Leandro KC, Conte-Junior CA (2017). *Lactococcus lactis* ssp. cremoris MRS47, a potential probiotic strain isolated from kefir grains, increases cis-9, trans-11-CLA and PUFA contents in fermented milk. J Funct Foods..

[22] Teles T, Ornellas RM, Borges Arcucio L, Messias Oliveira M, Nicoli JR, Villela Dias C, Trovatti Uetanabaro AP, Vinderola G (2016). Characterization of lactobacilli strains derived from cocoa fermentation in the south of Bahia for the development of probiotic cultures. LWT Food Sci Technol.

[23] Khan MI, Arshad MS, Anjum FM, Sameen A, Aneeq ur R, Gill WT (2011). Meat as a functional food with special reference to probiotic sausages. Food Res Int.

[24] Delbarre-Ladrat C, Chéret R, Taylor R, Verrez-Bagnis V (2006). Trends in postmortem aging in fish: understanding of proteolysis and disorganization of the myofibrillar structure. Crit Rev Food Sci Nutr.

[25] Benbrook CM, Benbrook CM (2005). Elevating antioxidant levels in food through organic farming and food processing. State of science review: elevating antioxidant levels in food.

[26] Leroy F, Verluyten J, De Vuyst L (2006). Functional meat starter cultures for improved sausage fermentation. Int J Food Microbiol.

[27] Ayyash M, Al-Nuaimi AK, Al-Mahadin S, Liu S-Q (2018). In vitro investigation of anticancer and ACE-inhibiting activity, α-amylase and α-glucosidase inhibition, and antioxidant activity of camel milk fermented with camel milk probiotic: a comparative study with fermented bovine milk. Food Chem.

[28] da Gomes Cruz A, Buriti FCA, de Batista Souza CH, Fonseca Faria JA, Isay Saad SM (2009). Probiotic cheese: health benefits, technological and stability aspects. Trends Food Sci Technol..

[29] Picot L, Bordenave S, Didelot S, Fruitier-Arnaudin I, Sannier F, Thorkelsson G, Bergé J, Guérard F, Chabeaud A, Piot J (2006). Antiproliferative activity of fish protein hydrolysates on human breast cancer cell lines. Process Biochem.

[30] Saelim K, Jampaphaeng K, Maneerat S (2017). Functional properties of Lactobacillus plantarum S0/7 isolated fermented stinky bean (Sa Taw Dong) and its use as a starter culture. J Funct Foods.

[31] Charteris WP, Kelly PM, Morelli L, Collins JK (1998). Antibiotic susceptibility of potentially probiotic Lactobacillus species. J Food Prot.

[32] Angmo K, Kumari A (2016). Savitri, Bhalla TC: Probiotic characterization of lactic acid bacteria isolated from fermented foods and beverage of Ladakh. LWT Food Sci Technol.

[33] Mishra V, Prasad DN (2005). Application of in vitro methods for selection of *Lactobacillus casei* strains as potential probiotics. Int J Food Microbiol.

[34] World Health Organization (WHO). WHO estimates of the global burden of foodborne diseases: foodborne disease burden epidemiology reference group 2007–2015. http://apps.who.int/iris/bitstream/handle/10665/199350/9789241565165_eng.pdf. Geneva: Switzerland: World Health Organization; 2015.

[35] Zuo F, Yu R, Feng X, Chen L, Zeng Z, Khaskheli GB, Ma H, Chen S (2016). Characterization and in vitro properties of potential probiotic *Bifidobacterium* strains isolated from breast-fed infant feces. Ann Microbiol.

[36] Collado MC, Meriluoto J, Salminen S (2008). Adhesion and aggregation properties of probiotic and pathogen strains. Eur Food Res Technol.

[37] Liong MT, Shah NP (2005). Bile salt deconjugation ability, bile salt hydrolase activity and cholesterol co-precipitation ability of lactobacilli strains. Int Dairy J.

[38] Vizoso Pinto MG, Franz CM, Schillinger U, Holzapfel WH (2006). Lactobacillus spp with in vitro probiotic properties from human faeces and traditional fermented products. Int J Food Microbiol..

[39] Sachindra NM, Mahendrakar NS (2010). Stability of carotenoids recovered from shrimp waste and their use as colorant in fish sausage. J Food Sci Technol.

[40] Mejri L, Ziadi A, El Adab S, Boulares M, Essid I, Hassouna M (2017). Effect of commercial starter cultures on physicochemical, microbiological and textural characteristics of a traditional dry fermented sausage reformulated with camel meat and hump fat. J Food Meas Charact.

[41] Berardo A, De Maere H, Stavropoulou DA, Rysman T, Leroy F, De Smet S (2016). Effect of sodium ascorbate and sodium nitrite on protein and lipid oxidation in dry fermented sausages. Meat Sci.

[42] Van Ba H, Seo HW, Cho SH, Kim YS, Kim JH, Ham JS, Park BY, Pil-Nam S (2017). Effects of extraction methods of shiitake by-products on their antioxidant and antimicrobial activities in fermented sausages during storage. Food Control.

[43] Sah BNP, Vasiljevic T, McKechnie S, Donkor ON (2014). Effect of probiotics on antioxidant and antimutagenic activities of crude peptide extract from yogurt. Food Chem.

[44] Nielsen P, Petersen D, Dambmann C (2001). Improved method for determining food protein degree of hydrolysis. J Food Sci.

[45] Kim YM, Wang MH, Rhee HI (2004). A novel α-glucosidase inhibitor from pine bark. Carbohydr Res.

[46] Elfahri KR, Vasiljevic T, Yeager T, Donkor ON (2016). Anti-colon cancer and antioxidant activities of bovine skim milk fermented by selected *Lactobacillus helveticus* strains. J Dairy Sci.

