# Supplementary material for: Isolation, identification, and potential probiotic characterization of isolated lactic acid bacteria and in vitro investigation of the cytotoxicity, antioxidant, and antidiabetic activities in fermented sausage
Source: Microb Cell Fact. 2019 Nov 5;18:188. doi: 10.1186/s12934-019-1239-1 (PMC6833168; doi:10.1186/s12934-019-1239-1)
Supplement: Supplementary file 1 — Additional file 1. Additional tables. [file 12934_2019_1239_MOESM1_ESM.docx]

**Additional file**

**Isolation, Identification, and Probiotic Characterization of Isolated Lactic Acid Bacteria and in-vitro Investigation of the Cytotoxicity, Antioxidant, and Antidiabetic Activities in Fermented Sausage**

**Nadia S. AlKalbani^1^, Mark S. Turner^2^, Mutamed Ayyash^1*^**

^1^ Food Science Department, College of Food and Agriculture, United Arab Emirates University (UAEU), PO Box 1555, Al Ain, UAE

^2^ School of Agriculture and Food Sciences, The University of Queensland (UQ), Brisbane, Australia

**^1^ Corresponding author:**

Dr. Mutamed Ayyash

Food Science Department

College of Food & Agriculture

United Arab Emirates University (UAEU)

T: +971 3 713 4552

F: +971 3 767 5336

E: [mutamed.ayyash@uaeu.ac.ae](mailto:mutamed.ayyash@uaeu.ac.ae)

Table S1: Gastric and intestinal tolerances of 85 isolates (Log_10_ CFU/mL)

| Isolate | Sample code | Gastric tolerance (pH 2.0 + pepsin) | | Intestinal tolerance (pH 8.0 + Trypsin) | |
| --- | --- | --- | --- | --- | --- |
|  |  | 0 h | 2 h | 0 h | 6 h |
| Iso1 | 06M | 6.64±0.14^1^ | 3.28±0.34 | 6.6±0.19 | 6.4±0.07 |
| Iso2 | 09M | 7.41±0.14 | 2.55±0.57 | 7.1±0.07 | 6.6±0.16 |
| Iso3 | 09MS | 7.22±0.22 | 3.63±0.55 | 7.2±0.21 | 6.9±0.08 |
| Iso4 | 11M | 7.15±0.18 | 6.46±0.27 | 7.1±0.08 | 7.0±0.14 |
| Iso5 | 14M | 6.73±0.24 | 3.51±0.27 | 6.7±0.45 | 6.6±0.17 |
| Iso6 | 17M | 7.36±0.21 | 6.52±0.11 | 7.5±0.06 | 6.6±0.13 |
| Iso7 | 19MS | 7.38±0.15 | 6.29±0.15 | 7.4±0.07 | 6.6±0.13 |
| Iso8 | 20MS | 6.43±0.45 | 5.19±0.16 | 7.4±0.07 | 6.6±0.13 |
| Iso9 | 21MS | 7.56±0.18 | 2.21±0.13 | 7.4±0.07 | 6.8±0.09 |
| Iso10 | 25MS | 6.68±0.39 | 2.22±0.11 | 7.4±0.07 | 7.0±0.16 |
| Iso11 | 27M | 7.08±0.13 | 4.19±0.09 | 7.3±0.17 | 6.8±0.12 |
| Iso12 | 27MS | 6.32±0.28 | 2.38±0.11 | 6.4±0.26 | 6.4±0.19 |
| Iso13 | 31MS | 7.62±0.27 | 5.15±0.41 | 7.1±0.70 | 7.2±0.00 |
| Iso14 | 32M | 6.91±0.31 | 3.54±0.25 | 6.6±0.11 | 6.2±0.22 |
| Iso15 | 34M | 7.59±0.18 | 2.06±0.08 | 7.3±0.39 | 7.1±0.11 |
| Iso16 | 36MS | 7.61±0.22 | 2.31±0.23 | 6.6±0.10 | 6.6±0.18 |
| Iso17 | 37M | 7.06±0.14 | 4.22±0.04 | 7.2±0.20 | 7.2±0.10 |
| Iso18 | 41M | 7.35±0.11 | 5.86±0.38 | 6.6±0.03 | 6.5±0.17 |
| Iso19 | 42M | 7.29±0.09 | 6.16±0.05 | 7.3±0.25 | 7.2±0.26 |
| Iso20 | 44M | 7.24±0.09 | 5.44±0.09 | 7.2±0.04 | 6.7±0.11 |
| Iso21 | 46MS | 7.74±0.09 | 7.08±0.06 | 7.6±0.00 | 6.7±0.22 |
| Iso22 | 49MS | 7.73±0.09 | 3.25±0.05 | 7.4±0.06 | 6.7±0.21 |
| Iso23 | 53M | 7.40±0.12 | 2.34±0.37 | 7.1±0.14 | 6.5±0.04 |
| Iso24 | 53MS | 7.62±0.08 | 3.77±0.08 | 7.5±0.04 | 7.2±0.13 |
| Iso25 | 59M | 7.53±0.02 | 3.57±0.18 | 7.3±0.11 | 6.6±0.04 |
| Iso26 | 62M | 7.35±0.10 | 6.12±0.08 | 7.4±0.17 | 6.2±0.26 |
| Iso27 | 66MS | 7.47±0.02 | 3.60±0.26 | 7.4±0.13 | 6.5±0.04 |
| Iso28 | 67MS | 6.83±0.66 | 2.17±0.18 | 7.4±0.05 | 7.1±0.07 |
| Iso29 | 70MS | 7.55±0.12 | 2.28±0.03 | 7.4±0.10 | 6.5±0.09 |
| Iso30 | 71MS | 7.52±0.02 | 5.95±0.01 | 6.9±0.82 | 7.1±0.36 |
| Iso31 | 72MS | 7.30±0.13 | 2.19±0.16 | 7.2±0.02 | 6.9±0.42 |
| Iso32 | 75M | 7.49±0.04 | 2.26±0.31 | 7.2±0.09 | 7.2±0.31 |
| Iso33 | 75MS | 7.42 ±0.05 | 2.21±0.13 | 7.4±0.23 | 6.9±0.63 |
| Iso34 | 77MS | 7.41±0.02 | 3.00±0.32 | 7.3±0.11 | 7.2±0.27 |
| Iso35 | 78MS | 7.50±0.11 | 2.21±0.13 | 7.3±0.17 | 6.8±0.57 |
| Iso36 | 79MS | 7.44±0.08 | 6.26±0.02 | 7.3±0.06 | 7.2±0.29 |
| Iso37 | 84M | 6.71±0.23 | 5.86±0.13 | 6.7±0.20 | 6.5±0.08 |
| Iso38 | 07B | 7.59±0.03 | 3.70±0.08 | 7.4±0.01 | 6.3±0.38 |
| Iso39 | 07BS | 7.43±0.19 | 2.21±0.13 | 7.4±0.01 | 6.5±0.03 |
| Iso40 | 08B | 7.48±0.05 | 3.16±0.02 | 7.4±0.21 | 6.6±0.12 |
| Iso41 | 09B | 7.44±0.04 | 2.49±0.16 | 6.7±0.57 | 6.6±0.12 |
| Iso42 | 13B | 7.42±0.12 | 2.63±0.21 | 7.3±0.11 | 6.4±0.08 |
| Iso43 | 13BS | 7.45±0.07 | 3.31±0.23 | 7.2±0.03 | 6.4±0.11 |
| Iso44 | 14B | 7.52±0.02 | 3.40±0.15 | 7.4±0.04 | 6.3±0.32 |
| Iso45 | 14BS | 7.54±0.04 | 3.41±0.57 | 7.3±0.01 | 6.4±0.08 |
| Iso46 | 16B | 7.55±0.12 | 3.45±0.21 | 7.1±0.52 | 6.4±0.19 |
| Iso47 | 16BS | 7.64±0.11 | 4.20±0.31 | 7.4±0.08 | 6.4±0.13 |
| Iso48 | 25B | 7.66±0.05 | 3.39±0.55 | 7.4±0.23 | 6.4±0.16 |
| Iso49 | 27B | 7.87±0.03 | 5.70 ±0.13 | 7.6±0.28 | 6.4±0.14 |
| Iso50 | 27BS | 7.52±0.13 | 2.21±0.13 | 7.5±0.26 | 6.3±0.07 |
| Iso51 | 34B | 7.48±0.11 | 6.46±0.26 | 7.4±0.09 | 6.4±0.11 |
| Iso52 | 39B | 7.22±0.16 | 6.18±0.08 | 7.2±0.22 | 6.3±0.09 |
| Iso53 | 40B | 7.39±0.01 | 3.27±0.28 | 7.2±0.06 | 6.3±0.10 |
| Iso54 | 42B | 7.51±0.09 | 5.92±0.37 | 7.5±0.18 | 6.3±0.09 |
| Iso55 | 45B | 7.44±0.05 | 5.90±0.24 | 7.5±0.11 | 6.3±0.09 |
| Iso56 | 47BS | 7.41±0.07 | 3.29±0.16 | 7.4±0.18 | 6.3±0.09 |
| Iso57 | 49B | 7.43±0.04 | 5.52±0.15 | 7.6±0.14 | 7.6±0.14 |
| Iso58 | 51B | 7.37±0.13 | 2.19±0.16 | 7.6±0.14 | 7.6±0.14 |
| Iso59 | 52B | 7.22±0.07 | 2.28±0.28 | 7.5±0.03 | 7.6±0.27 |
| Iso60 | 52BS | 7.30±0.04 | 3.03±0.03 | 7.3±0.04 | 7.3±0.03 |
| Iso61 | 53B | 7.50±0.16 | 2.37±0.32 | 7.7±0.16 | 7.5±0.13 |
| Iso62 | 54B | 7.17±0.12 | 2.32±0.03 | 7.6±0.15 | 7.6±0.14 |
| Iso63 | 54BS | 7.49±0.02 | 6.12±0.35 | 7.6±0.18 | 7.6±0.23 |
| Iso64 | 57BS | 7.26±0.19 | 5.99±0.29 | 7.3±0.06 | 7.3±0.26 |
| Iso65 | 58B | 7.35±0.07 | 2.22±0.11 | 7.0±0.12 | 7.2±0.11 |
| Iso66 | 61B | 7.50±0.07 | 2.19±0.16 | 7.1±0.01 | 7.1±0.06 |
| Iso67 | 66B | 6.83±0.13 | 6.10±0.02 | 6.7±0.05 | 7.2±0.38 |
| Iso68 | 70BS | 7.34±0.10 | 6.62±0.13 | 7.5±0.01 | 7.4±0.21 |
| Iso69 | 71B | 7.36±0.21 | 2.19±0.16 | 7.5±0.10 | 7.5±0.15 |
| Iso70 | 71BS | 7.36±0.08 | 2.24±0.09 | 7.3±0.03 | 7.3±0.15 |
| Iso71 | 72B | 7.16±0.06 | 2.19±0.16 | 7.3±0.08 | 7.2±0.04 |
| Iso72 | 72BS | 7.45±0.02 | 2.28±0.28 | 7.3±0.06 | 7.3±0.11 |
| Iso73 | 73BS | 7.69±0.09 | 2.28±0.28 | 7.3±0.09 | 7.3±0.10 |
| Iso74 | 74BS | 7.31±0.07 | 6.19±0.10 | 7.6±0.32 | 7.6±0.11 |
| Iso75 | 75B | 7.81±0.06 | 5.83±0.10 | 7.6±0.23 | 7.7±0.43 |
| Iso76 | 75BS | 7.79±0.16 | 2.91±0.33 | 7.8±0.19 | 7.7±0.15 |
| Iso77 | 76B | 7.24±0.12 | 2.28±0.28 | 7.8±0.17 | 7.8±0.16 |
| Iso78 | 77B | 7.15 ±0.19 | 2.31±0.01 | 7.4±0.18 | 7.4±0.44 |
| Iso79 | 79B | 7.59±0.03 | 6.09±0.30 | 7.6±0.15 | 7.6±0.05 |
| Iso80 | 80B | 7.34±0.06 | 2.36±0.35 | 7.6±0.10 | 7.6±0.07 |
| Iso81 | 81B | 6.62±0.27 | 3.89±0.02 | 7.0±0.21 | 7.1±0.04 |
| Iso82 | 81BS | 7.36±0.12 | 5.84±0.07 | 7.1±0.25 | 7.3±0.15 |
| Iso83 | 83BS | 7.37±0.11 | 6.21±0.05 | 7.5±0.14 | 7.5±0.10 |
| Iso84 | 84B | 7.25±0.04 | 6.66±0.07 | 7.2±0.02 | 7.1±0.05 |
| Iso85 | 84BS | 7.36±0.08 | 2.37±0.46 | 7.2±0.03 | 7.1±0.04 |

^1^Values are mean ± standard error of duplicate

Table S2: Bile suppression (%) of lactic acid bacteria isolates at 3 and 6 h.

| Isolate | Sample code | Bile suppression (%) | |  |  |  |  |
| --- | --- | --- | --- | --- | --- | --- | --- |
|  |  | 1.0% Oxgall | | 0.3% Cholic acid | | 1.0% Taurocholic acid | |
|  |  | 3 h | 6 h | 3 h | 6 h | 3 h | 6 h |
| Iso1 | 06M | 24.3±1.5^1^ | 52.0±1.1 | 10.3±1.4 | 7.2±1.2 | 4.4±0.6 | 24.7±5.8 |
| Iso2 | 09M | 36.0±4.6 | 50.6±2.9 | 0.0±0.0 | 0.0±0.0 | 0.0±0.0 | 0.0±0.0 |
| Iso3 | 09MS | 44.3±1.9 | 51.4±4.7 | 0.0±0.0 | 0.0±0.0 | 5.9±2.1 | 0.0±0.0 |
| Iso4 | 11M | 43.6±1.2 | 62.4±4.0 | 0.0±0.0 | 0.0±0.0 | 9.2±1.5 | 35.0±2.3 |
| Iso5 | 14M | 36.1±9.8 | 61.0±6.7 | 0.0±0.0 | 0.0±0.0 | 5.5±0.8 | 0.0±0.0 |
| Iso6 | 17M | 18.0±0.9 | 44.8±5.9 | 0.0±0.0 | 0.0±0.0 | 11.3±5.7 | 0.0±0.0 |
| Iso7 | 19MS | 6.8±1.3 | 23.0±7.1 | 3.8±0.4 | 0.0±0.0 | 1.7±0.6 | 0.0±0.0 |
| Iso8 | 20MS | 25.3±0.7 | 56.2±2.6 | 0.0±0.0 | 5.4±0.8 | 8.3±2.7 | 19.1±3.4 |
| Iso9 | 21MS | 46.3±5.5 | 63.2±5.7 | 4.2±1.1 | 0.0±0.0 | 12.8±0.9 | 0.0±0.0 |
| Iso10 | 25MS | 7.2±1.5 | 28.9±2.7 | 0.0±0.0 | 1.5±0.4 | 3.3±0.3 | 20.4±2.5 |
| Iso11 | 27M | 31.2±4.1 | 49.4±5.2 | 0.0±0.0 | 0.0±0.0 | 2.0±0.2 | 0.0±0.0 |
| Iso12 | 27MS | 0.0±0.0 | 18.1±2.0 | 0.0±0.0 | 1.4±0.2 | 5.8±0.3 | 19.6±0.3 |
| Iso13 | 31MS | 45.5±1.4 | 59.1±1.0 | 2.9±0.1 | 0.0±0.0 | 11.9±0.4 | 0.0±0.0 |
| Iso14 | 32M | 11.3±0.8 | 31.0±1.9 | 0.0±0.0 | 7.8±0.6 | 7.4±2.9 | 23.3±4.9 |
| Iso15 | 34M | 33.3±3.0 | 63.8±0.5 | 2.8±0.2 | 0.0±0.0 | 10.8±2.4 | 9.3±0.7 |
| Iso16 | 36MS | 35.1±2.2 | 39.1±1.2 | 0.0±0.0 | 0.0±0.0 | 3.4±1.6 | 0.0±0.0 |
| Iso17 | 37M | 40.9±0.1 | 60.2±2.1 | 17.6±0.2 | 0.0±0.0 | 12.0±1.0 | 0.0±0.0 |
| Iso18 | 41M | 16.2±1.2 | 28.9±6.2 | 4.0±1.5 | 5.7±5.1 | 8.5±2.0 | 17.0±7.5 |
| Iso19 | 42M | 28.8±3.9 | 38.2±8.0 | 0.7±0.4 | 12.0±1.4 | 12.0±1.1 | 13.2±2.3 |
| Iso20 | 44M | 34.1±1.2 | 49.2±2.8 | 10.3±0.2 | 5.6±2.0 | 6.7±3.0 | 0.0±0.0 |
| Iso21 | 46MS | 33.8±0.5 | 57.1±0.9 | 0.1±0.1 | 0.0±0.0 | 2.4±0.3 | 3.6±0.8 |
| Iso22 | 49MS | 39.9±0.9 | 46.2±4.2 | 6.9±1.1 | 0.0±0.0 | 14.1±2.8 | 2.9±0.4 |
| Iso23 | 53M | 42.1±0.6 | 57.7±3.7 | 0.0±0.0 | 0.0±0.0 | 8.4±0.4 | 0.0±0.0 |
| Iso24 | 53MS | 41.7±1.3 | 54.5±0.7 | 0.0±0.0 | 0.0±0.0 | 3.3±0.1 | 0.0±0.0 |
| Iso25 | 59M | 35.8±1.7 | 38.4±0.7 | 3.3±0.4 | 0.0±0.0 | 5.8±0.3 | 0.0±0.0 |
| Iso26 | 62M | 39.3±0.8 | 51.6±1.1 | 0.0±0.0 | 0.0±0.0 | 1.4±0.4 | 0.0±0.0 |
| Iso27 | 66MS | 39.6±1.5 | 58.4±3.3 | 6.5±2.0 | 0.0±0.0 | 10.2±0.6 | 0.0±0.0 |
| Iso28 | 67MS | 40.9±1.5 | 53.6±0.2 | 0.0±0.0 | 0.0±0.0 | 11.2±0.1 | 0.0±0.0 |
| Iso29 | 70MS | 40.6±1.9 | 61.3±0.6 | 3.0±0.9 | 0.0±0.0 | 4.8±1.7 | 0.0±0.0 |
| Iso30 | 71MS | 37.7±1.4 | 48.3±0.7 | 4.6±0.5 | 0.0±0.0 | 2.8±0.2 | 0.0±0.0 |
| Iso31 | 72MS | 45.1±4.9 | 52.5±0.8 | 0.0±0.0 | 0.0±0.0 | 3.4±0.1 | 0.0±0.0 |
| Iso32 | 75M | 31.1±2.3 | 54.1±0.8 | 0.0±0.0 | 0.0±0.0 | 6.8±0.5 | 1.7±0.2 |
| Iso33 | 75MS | 38.1±0.5 | 52.7±4.8 | 5.1±1.2 | 0.0±0.0 | 10.4±1.8 | 0.0±0.0 |
| Iso34 | 77MS | 35.7±4.4 | 51.9±0.7 | 3.8±0.1 | 0.0±0.0 | 11.8±0.6 | 0.0±0.0 |
| Iso35 | 78MS | 35.6±1.1 | 53.3±2.3 | 0.0±0.0 | 0.0±0.0 | 7.2±0.2 | 3.4±0.0 |
| Iso36 | 79MS | 41.9±2.1 | 59.0±2.5 | 1.5±0.1 | 0.0±0.0 | 12.2±1.1 | 0.0±0.0 |
| Iso37 | 84M | 38.6±0.8 | 57.2±1.1 | 3.3±0.5 | 0.7±0.1 | 10.0±2.3 | 1.7±2.4 |
| Iso38 | 07B | 34.4±0.8 | 40.0±2.5 | 0.2±0.0 | 0.0±0.0 | 3.3±0.9 | 0.0±0.0 |
| Iso39 | 07BS | 31.9±0.7 | 31.0±1.6 | 0.0±0.0 | 0.0±0.0 | 10.3±0.7 | 1.9±0.0 |
| Iso40 | 08B | 32.3±2.0 | 32.5±2.3 | 7.6±0.5 | 0.0±0.0 | 2.5±0.4 | 0.0±0.0 |
| Iso41 | 09B | 50.1±0.6 | 55.4±3.2 | 0.3±0.0 | 0.0±0.0 | 6.1±1.6 | 0.0±0.0 |
| Iso42 | 13B | 31.8±2.2 | 41.9±3.7 | 2.3±0.8 | 0.0±0.0 | 3.6±1.3 | 3.7±0.2 |
| Iso43 | 13BS | 53.8±1.2 | 54.3±2.2 | 13.2±1.1 | 0.3±0.4 | 3.6±5.1 | 0.0±0.0 |
| Iso44 | 14B | 35.2±2.9 | 34.4±1.3 | 5.6±1.5 | 0.0±0.0 | 6.6±0.0 | 0.0±0.0 |
| Iso45 | 14BS | 32.7±1.2 | 33.3±5.8 | 1.4±0.2 | 0.0±0.0 | 5.3±0.9 | 0.0±0.0 |
| Iso46 | 16B | 37.7±3.5 | 49.4±3.6 | 0.6±0.3 | 0.0±0.0 | 7.4±2.3 | 0.0±0.0 |
| Iso47 | 16BS | 40.2±2.2 | 51.3±1.5 | 1.4±0.7 | 0.0±0.0 | 7.9±1.4 | 0.0±0.0 |
| Iso48 | 25B | 25.9±1.1 | 50.7±2.4 | 7.4±0.8 | 4.8±2.2 | 11.9±2.1 | 8.9±0.2 |
| Iso49 | 27B | 40.0±1.6 | 62.1±1.0 | 6.1±1.5 | 2.4±0.3 | 13.9±1.4 | 7.4±2.1 |
| Iso50 | 27BS | 41.8±2.2 | 57.0±4.8 | 4.3±0.6 | 0.0±0.0 | 9.4±3.4 | 0.0±0.0 |
| Iso51 | 34B | 31.1±2.3 | 61.4±3.5 | 1.9±0.1 | 0.0±0.0 | 11.3±0.6 | 6.7±0.2 |
| Iso52 | 39B | 31.2±2.1 | 60.2±2.7 | 1.8±0.1 | 1.4±0.6 | 3.0±3.4 | 1.9±0.5 |
| Iso53 | 40B | 35.3±0.2 | 45.0±1.5 | 0.0±0.0 | 0.0±0.0 | 5.1±2.1 | 8.0±0.1 |
| Iso54 | 42B | 17.0±2.9 | 31.6±1.4 | 2.4±0.5 | 3.7±1.1 | 6.3±1.7 | 12.4±3.2 |
| Iso55 | 45B | 40.8±0.6 | 56.9±4.3 | 6.0±1.5 | 0.0±0.0 | 13.0±2.9 | 0.0±0.0 |
| Iso56 | 47BS | 43.6±14.2 | 58.6±0.9 | 3.5±1.0 | 1.1±1.6 | 7.6±0.1 | 0.6±0.9 |
| Iso57 | 49B | 35.3±0.5 | 50.3±0.9 | 1.5±0.1 | 0.0±0.0 | 10.4±0.7 | 1.1±0.1 |
| Iso58 | 51B | 32.8±4.7 | 44.8±1.2 | 0.0±0.0 | 7.8±0.1 | 0.0±0.0 | 7.2±0.4 |
| Iso59 | 52B | 35.7±0.0 | 49.8±4.1 | 0.0±0.0 | 0.0±0.0 | 4.7±0.2 | 0.0±0.0 |
| Iso60 | 52BS | 33.8±2.3 | 39.9±3.2 | 0.0±0.0 | 0.0±0.0 | 3.6±0.6 | 0.0±0.0 |
| Iso61 | 53B | 32.0±4.0 | 42.0±3.9 | 0.0±0.0 | 0.0±0.0 | 0.0±0.0 | 0.0±0.0 |
| Iso62 | 54B | 18.8±0.4 | 31.2±8.3 | 1.1±0.2 | 0.0±0.0 | 6.8±2.7 | 1.1±1.5 |
| Iso63 | 54BS | 33.6±3.2 | 49.2±2.5 | 1.3±0.2 | 0.0±0.0 | 8.8±1.3 | 0.0±0.0 |
| Iso64 | 57BS | 26.1±1.9 | 55.1±1.4 | 5.3±1.0 | 5.2±0.5 | 2.6±0.3 | 0.0±0.0 |
| Iso65 | 58B | 38.3±0.6 | 56.5±0.9 | 0.0±0.0 | 0.0±0.0 | 0.0±0.0 | 0.0±0.0 |
| Iso66 | 61B | 33.9±5.8 | 50.1±3.0 | 1.7±0.2 | 0.0±0.0 | 0.0±0.0 | 0.0±0.0 |
| Iso67 | 66B | 10.7±0.1 | 37.7±1.1 | 3.7±0.0 | 8.9±1.2 | 2.2±1.4 | 19.3±2.3 |
| Iso68 | 70BS | 32.9±2.0 | 40.9±0.9 | 3.1±0.5 | 0.0±0.0 | 11.2±1.3 | 0.0±0.0 |
| Iso69 | 71B | 35.3±3.0 | 47.7±4.5 | 0.0±0.0 | 0.0±0.0 | 0.8±0.6 | 0.0±0.0 |
| Iso70 | 71BS | 36.3±0.9 | 48.0±1.6 | 0.0±0.0 | 0.0±0.0 | 0.0±0.0 | 0.0±0.0 |
| Iso71 | 72B | 41.0±2.1 | 54.0±0.3 | 4.7±1.0 | 0.0±0.0 | 11.0±0.8 | 0.0±0.0 |
| Iso72 | 72BS | 37.3±3.0 | 57.2±2.0 | 2.2±0.1 | 0.0±0.0 | 15.0±1.2 | 11.7±0.9 |
| Iso73 | 73BS | 35.1±3.5 | 58.2±2.0 | 2.8±0.3 | 0.0±0.0 | 11.0±3.2 | 2.7±0.7 |
| Iso74 | 74BS | 32.6±1.3 | 44.4±1.3 | 4.6±0.0 | 0.0±0.0 | 10.6±0.1 | 0.0±0.0 |
| Iso75 | 75B | 39.7±3.2 | 63.1±1.6 | 7.7±2.1 | 4.5±0.6 | 15.8±1.0 | 14.2±2.0 |
| Iso76 | 75BS | 35.8±3.7 | 60.5±0.2 | 4.2 ±0.6 | 1.8±1.0 | 15.6±2.8 | 13.0±3.5 |
| Iso77 | 76B | 51.9±15.7 | 62.6±5.4 | 5.0±1.1 | 0.0±0.0 | 8.5±0.4 | 7.5±1.7 |
| Iso78 | 77B | 15.6±1.1 | 57.3±0.1 | 8.1±1.0 | 1.2±0.4 | 11.6±0.1 | 12.7±0.0 |
| Iso79 | 79B | 60.7±1.4 | 58.2±0.5 | 2.4±0.1 | 0.0±0.0 | 4.9±0.9 | 0.0±0.0 |
| Iso80 | 80B | 35.3±0.4 | 45.1±0.4 | 0.0±0.0 | 0.0±0.0 | 3.6±1.2 | 0.0±0.0 |
| Iso81 | 81B | 8.8±0.4 | 29.0±0.1 | 7.1±1.3 | 5.0±0.6 | 6.9±1.0 | 24.0±3.8 |
| Iso82 | 81BS | 28.4±4.1 | 38.6±1.5 | 0.0±0.0 | 0.0±0.0 | 1.7±0.9 | 0.0±0.0 |
| Iso83 | 83BS | 41.3±0.1 | 53.6±0.7 | 4.6±0.8 | 0.0±0.0 | 6.7±1.5 | 0.6±0.3 |
| Iso84 | 84B | 31.3±4.0 | 40.4±4.1 | 0.0±0.0 | 0.0±0.0 | 1.8±0.1 | 0.0±0.0 |
| Iso85 | 84BS | 22.4±1.1 | 20.4±0.5 | 0.0±0.0 | 0.0±0.0 | 0.0±0.0 | 0.0±0.0 |

^1^Values are mean ± standard error of duplicates

Table S3: Identified LAB isolates by 16S rDNA gene sequencing and Genbank accession numbers

| Isolate | Bacteria | Accession numbers |
| --- | --- | --- |
| 11M | *Enterococcus faecalis* | MF067467 |
| 17M | *Enterococcus faecalis* | MF067469 |
| 19MS | *Enterococcus faecium* | MF067470 |
| 46MS | *Enterococcus faecium* | MF067487 |
| 62M | *Enterococcus faecium* | MF067495 |
| 71MS | *Enterococcus faecalis* | MF067500 |
| 79MS | *Enterococcus faecium* | MF067509 |
| 39B | *Enterococcus faecium* | KY962871 |
| 45B | *Enterococcus faecium* | KY962874 |
| 54BS | *Enterococcus durans* | KY962882 |
| 57BS | *Enterococcus faecium* | KY962883 |
| 70BS | *Enterococcus durans* | KY962888 |
| 84B | *Enterococcus faecalis* | KY962905 |

Table S4: Prevalence of virulence genes in the 13 *Enterococcus* spp.

| Bacteria | Target gene^1^ | | | | | | | | |
| --- | --- | --- | --- | --- | --- | --- | --- | --- | --- |
|  | *cylL*_L_ | *cylL*_S_ | *asa*l | *gel*E | *esp* | *hyl* | *efa*A_fs_ | *agg* | *ace* |
| *E. faecalis* MF067467 | -^2^ | - | - | + | - | - | + | + | + |
| *E. faecalis* MF067469 | - | - | - | + | - | - | + | + | + |
| *E. faecium* MF067470 | - | - | - | - | - | - | - | + | - |
| *E. faecium* MF067487 | - | - | - | - | - | - | - | + | - |
| *E. faecium* MF067495 | - | - | - | - | - | - | - | + | - |
| *E. faecalis* MF067500 | - | - | - | + | - | - | + | + | + |
| *E. faecium* MF067509 | - | - | - | - | - | - | - | + | + |
| *E. faecium* KY962871 | - | - | - | - | - | - | - | + | + |
| *E. faecium* KY962874 | - | - | - | + | - | - | - | + | + |
| *E. durans* KY962882 | - | - | - | + | - | - | + | + | + |
| *E. faecium* KY962883 | - | - | - | + | - | - | + | + | + |
| *E. durans* KY962888 | - | - | - | + | - | - | + | + | + |
| *E. faecalis* KY962905 | - | - | - | + | - | - | + | + | + |

^1^ *cyl*L_L_ and *cyl*L_s_ (cytolisin structural subunits); *asa*1 (aggregation substance); *gel*E (gelatinase); *esp* (enterococcal surface protein); *hyl* (hyaluronidase); *efaA_fs_* (cell wall adhesion); *agg* (aggregation protein involved in adherence to eukaryotic cells); *ace* (adhesion of collagen protein)

^2^ (-) gene absent; (+) gene present

Table S5: Co-aggregation (%) of LAB with four foodborne pathogens during 4 h incubation at 37°C

| Incubation time | Bacteria | Co-aggregation (%) | |  |  |
| --- | --- | --- | --- | --- | --- |
|  |  | *E. coli* O157:H7 | *S.* Typhimurium | *L. monocytogenes* | *S. aureus* |
| 2 h | *E.faecalis* MF067467 | 13.0 ± 1.51^ab^ | 14.4 ± 3.37^a^ | 13.6 ± 4.81^a^ | 12.2 ± 4.63^a^ |
|  | *E.faecalis* MF067469 | 11.8 ± 1.96^abc^ | 13.6 ± 1.58^a^ | 12.1 ± 2.87^a^ | 11.5 ± 2.66^a^ |
|  | *E.faecium* MF067470 | 11.4 ± 4.72^abc^ | 13.3 ± 4.49^a^ | 12.0 ± 4.63^a^ | 12.2 ± 4.12^a^ |
|  | *E.faecium* MF067487 | 8.4 ± 2.82^abc^ | 11.6 ± 3.82^a^ | 9.7 ± 5.33^a^ | 9.5 ± 5.64^a^ |
|  | *E.faecium* MF067495 | 12.3 ± 3.67^abc^ | 15.3 ± 2.97^a^ | 13.7 ± 3.65^a^ | 13.4 ± 3.58^a^ |
|  | *E.faecalis* MF067500 | 12.8 ± 1.59^c^ | 14.2 ± 1.03^a^ | 10.8 ± 4.82^a^ | 10.8 ± 4.34^a^ |
|  | *E.faecium* MF067509 | 10.8 ± 2.30^c^ | 13.2 ± 3.18^a^ | 11.8 ± 4.48^a^ | 11.8 ± 3.45^a^ |
|  | *E.faecium* KY962871 | 10.5 ± 4.09^bc^ | 13.6 ± 4.39^a^ | 11.7 ± 4.78^a^ | 11.7 ± 4.31^a^ |
|  | *E.faecium* KY962874 | 8.9 ± 2.66^abc^ | 11.6 ± 3.24^a^ | 10.1 ± 4.47^a^ | 10.2 ± 5.11^a^ |
|  | *E.durans* KY962882 | 8.7 ± 1.93^a^ | 10.9 ± 1.60^a^ | 9.6 ± 2.64^a^ | 9.1 ± 2.80^a^ |
|  | *E.faecium* KY962883 | 9.8 ± 3.05^abc^ | 12.7 ± 2.66^a^ | 10.2 ± 4.07^a^ | 10.4 ± 4.76^a^ |
|  | *E.durans* KY962888 | 10.9 ± 2.66^abc^ | 14.7 ± 2.95^a^ | 12.7 ± 3.34^a^ | 12.4 ± 4.48^a^ |
|  | *E.faecalis* KY962905 | 11.7 ± 1.84^abc^ | 13.9 ± 2.55^a^ | 12.1 ± 3.72^a^ | 8.7 ± 9.23^a^ |
| 4 h | *E.faecalis* MF067467 | 18.4 ± 4.42^ab^ | 20.2 ± 4.28^bc^ | 18.7 ± 6.04^a^ | 18.4 ± 4.27^a^ |
|  | *E.faecalis* MF067469 | 18.8 ± 2.60^a^ | 18.8 ± 2.65^bc^ | 16.9 ± 1.32^a^ | 15.3 ± 2.22^ab^ |
|  | *E.faecium* MF067470 | 14.5 ± 5.03^abcd^ | 16.1 ± 5.49^c^ | 15.3 ± 5.21^a^ | 15.3 ± 3.97^ab^ |
|  | *E.faecium* MF067487 | 11.8 ± 4.16^abcd^ | 14.5 ± 4.92^bc^ | 12.4 ± 5.02^a^ | 11.9 ± 5.24^ab^ |
|  | *E.faecium* MF067495 | 15.2 ± 3.94^cd^ | 18.7 ± 2.31^abc^ | 16.4 ± 4.81^a^ | 16.2 ± 3.05^ab^ |
|  | *E.faecalis* MF067500 | 17.4 ± 2.34^d^ | 24.7 ± 9.99^c^ | 13.2 ± 5.24^a^ | 13.4 ± 4.05^ab^ |
|  | *E.faecium* MF067509 | 14.9 ± 3.55^cd^ | 33.2 ± 19.51^ab^ | 15.4 ± 4.09^a^ | 15.2 ± 3.34^ab^ |
|  | *E.faecium* KY962871 | 14.1 ± 3.87^bcd^ | 20.1 ± 0.36^ab^ | 15.1 ± 4.80^a^ | 15.0 ± 3.95^ab^ |
|  | *E.faecium* KY962874 | 12.0 ± 3.27^abcd^ | 23.5 ± 9.97^bc^ | 12.7 ± 4.54^a^ | 12.7 ± 4.91^ab^ |
|  | *E.durans* KY962882 | 11.9 ± 2.96^a^ | 28.3 ± 17.21^abc^ | 12.4 ± 4.10^a^ | 12.0 ± 3.82^ab^ |
|  | *E.faecium* KY962883 | 13.5 ± 4.58^abcd^ | 28.1 ± 14.98^a^ | 13.4 ± 5.28^a^ | 13.2 ± 4.45^ab^ |
|  | *E.durans* KY962888 | 15.6 ± 4.57^abc^ | 28.9 ± 11.80^ab^ | 16.2 ± 4.61^a^ | 15.8 ±5.30^b^ |
|  | *E.faecalis* KY962905 | 16.6 ± 2.16^abcd^ | 27.6 ± 10.91^ab^ | 15.3 ± 4.59^a^ | 11.5 ± 10.58^ab^ |

Values are mean ± standard deviation of triplicates

^a–d^ Mean values in the same column with different uppercase superscripts differ significantly (p < 0.05).

Table S6: Heat (60°C/5 min), lysozyme resistance (log_10_ CFU/ml) and exopolysaccharide production

| Bacteria | Heat resistance (log_10_ CFU/ml) | | Lysozyme resistance (log_10_ CFU/ml) | | Exopolysaccharide |
| --- | --- | --- | --- | --- | --- |
|  | 0 min | 5 min | 0 min | 90 min |  |
| *E. faecalis* MF067467 | 8.9 ± 0.0^fg^ | 7.7 ± 0.1^g^ | 9.2 ± 0.16^cd^ | 8.9 ± 0.01^d^ | + |
| *E. faecalis* MF067469 | 8.7 ± 0.1^g^ | 8.1 ± 0.1^f^ | 9.5 ± 0.08^ab^ | 9.1 ± 0.16^bcd^ | + |
| *E. faecium* MF067470 | 9.9 ± 0.1^bc^ | 9.1 ± 0.1^bc^ | 9.6 ± 0.07^a^ | 9.3 ± 0.05^a^ | + |
| *E. faecium* MF067487 | 9.9 ± 0.3^b^ | 9.5 ± 0.1^a^ | 9.5 ± 0.02^ab^ | 9.2 ± 0.05^abc^ | - |
| *E. faecium* MF067495 | 9.9 ± 0.2^bc^ | 9.5 ± 0.0^a^ | 9.5 ± 0.05^ab^ | 9.2 ± 0.11^a^ | + |
| *E. faecalis* MF067500 | 9.3 ± 0.2^de^ | 8.8 ± 0.1^d^ | 9.4 ± 0.06^bcd^ | 9.2 ± 0.10^abc^ | + |
| *E. faecium* MF067509 | 9.8 ± 0.2^bc^ | 9.2 ± 0.0^bc^ | 9.0 ± 0.13^e^ | 9.0 ± 0.15^cd^ | + |
| *E. faecium* KY962871 | 9.7 ± 0.2^bc^ | 9.3 ± 0.2^b^ | 9.3 ± 0.04^d^ | 9.1 ± 0.08^abcd^ | + |
| *E. faecium* KY962874 | 10.3 ± 0.2^a^ | 9.5 ± 0.1^a^ | 9.4 ± 0.06^bcd^ | 9.1± 0.18^ab^ | + |
| *E. durans* KY962882 | 9.8 ± 0.2^bc^ | 9.6 ± 0.0^a^ | 9.3 ± 0.11^bcd^ | 9.1 ± 0.20^ab^ | + |
| *E. faecium* KY962883 | 9.6 ± 0.2^cd^ | 9.0 ± 0.1^c^ | 9.3 ± 0.04^bcd^ | 8.8 ± 0.45^abcd^ | + |
| *E. durans* KY962888 | 9.9 ± 0.3^b^ | 9.5 ± 0.1^a^ | 9.2 ± 0.06^d^ | 9.0 ± 0.24^d^ | + |
| *E. faecalis* KY962905 | 9.1 ± 0.2^ef^ | 8.6 ± 0.0^e^ | 9.5 ± 0.01^abc^ | 9.0 ± 0.20^d^ | + |

Values are mean ± standard error of triplicates; (-): no EPS production

^a–f^ Mean values in the same column with different uppercase superscripts differ significantly (p < 0.05).

Table S7: Bacterial population, pH values, and TBAR in control and fermented sausages

| Storage period  (days) | Control | Commercial | *E. durans* K882 | *E. faecalis* K905 | *E. faecium* K874 | *E. faecium* M470 | *E. faecium* M495 | *E. faecium* M509 | |
| --- | --- | --- | --- | --- | --- | --- | --- | --- | --- |
|  | **Bacterial populations Log_10_ CFU/ml** | | | | | | | |  |
| 0 | 3.3±0.14^Ac^ | 7.4±0.54^Ab^ | 8.2±0.28^Ba^ | 8.7±0.07^Aa^ | 8.6±0.01^ABa^ | 8.5±0.62^Aa^ | 8.4±0.10^Aa^ | 8.4±0.09^Ba^ | |
| 7 | 3.3±0.04^Ae^ | 7.5±0.05^Ad^ | 8.3±0.10^ABcd^ | 8.6±0.14^Abc^ | 9.3±0.97^Aab^ | 7.9±0.09^Acd^ | 8.0±0.06^Ccd^ | 9.8±0.87^Aa^ | |
| 14 | 3.1±0.12^Ae^ | 7.6±0.03^Ad^ | 8.5±0.08^Aa^ | 8.2±0.07^Abc^ | 8.4±0.07^ABa^ | 8.1±0.25^Ac^ | 8.2±0.09^Bbc^ | 8.3±0.13^Bab^ | |
| 21 | 3.2±0.08^Ad^ | 7.4±0.09^Ac^ | 8.2±0.05^Bab^ | 8.6±0.75^Aa^ | 8.0±0.07^Bb^ | 8.0±0.18^Ab^ | 8.1±0.06^BCa^ | 7.7±0.28^Bbc^ | |
|  | **pH values** | | | | | | | |  |
| 0 | 6.7±0.07^Da^ | 6.6±0.07^Aab^ | 5.9±0.05^Acd^ | 5.8±0.58^Ad^ | 5.9±0.60^Ad^ | 6.2±0.02^Aabcd^ | 6.4±0.07^Aabc^ | 6.1±0.22^Abcd^ | |
| 7 | 7.6±0.04^Aa^ | 5.0±0.54^Bb^ | 4.6±0.02^Ac^ | 4.7±0.05^Bc^ | 4.7±0.02^Bc^ | 4.8±0.01^Cc^ | 4.7±0.02^Cc^ | 4.7±0.02^Bc^ | |
| 14 | 6.9±0.03^Ca^ | 4.9±0.02^Bb^ | 4.8±0.01^Be^ | 5.0±0.01^Bc^ | 4.7±0.01^Bg^ | 4.7±0.04^Df^ | 4.8±0.03^Bd^ | 4.8±0.02^Bde^ | |
| 21 | 7.3±0.09^Ba^ | 4.7±0.04^Bb^ | 4.7±0.02^Cf^ | 4.8±0.03^Bcd^ | 4.8±0.01^Bde^ | 4.8±0.02^Bc^ | 4.7±0.03^BCef^ | 4.7±0.03^Bf^ | |
|  | **TBAR (mg MDA/Kg)** | | | | | | | |  |
| 0 | 0.44±0.02^Cd^ | 0.50±0.01^Bc^ | 0.61±0.02^Aa^ | 0.60±0.01^Aa^ | 0.47±0.01^ABd^ | 0.46±0.01^Bd^ | 0.56±0.02^Ab^ | 0.41±0.02^Be^ | |
| 7 | 0.53±0.03^Bab^ | 0.41±0.05^Cde^ | 0.59±0.01^Aa^ | 0.49±0.07^Bbc^ | 0.41±0.02^Bde^ | 0.40±0.05^Bde^ | 0.44±0.00^Bcd^ | 0.37±0.04^Be^ | |
| 14 | 0.60±0.05^Aa^ | 0.49±0.04^Bb^ | 0.49±0.06^Bb^ | 0.50±0.05^Bb^ | 0.51±0.02^Ab^ | 0.41±0.02^Bcd^ | 0.47±0.03^Bbc^ | 0.39±0.03^Bd^ | |
| 21 | 0.47±0.02^Cd^ | 0.62±0.01^Aa^ | 0.63±0.05^Aa^ | 0.60±0.05^Aab^ | 0.51±0.06^Acd^ | 0.58±0.04^Aabc^ | 0.54±0.04^Abcd^ | 0.51±0.04^Abc^ | |

^a-e^ Mean values in the same row with different lowercase superscripts differ (*p* < 0.05).

^A-D^ Mean values in the same column with different uppercase superscript differs (*p* < 0.05).

Values are the mean ± standard deviation of n=3.

Table S8: Pearson’s correlations between parameters

|  | Log | pH | TBAR | DH% | ABTS% | DPPH% | Glucosidase | Amylase | Caco2 | MCF7 |
| --- | --- | --- | --- | --- | --- | --- | --- | --- | --- | --- |
| Log | 1.000 | -0.719 | -0.106 | 0.881** | 0.283** | 0.311** | 0.336** | 0.407** | 0.306** | 0.202* |
|  |  | *<0.001* | *0.304* | *<0.001* | *0.005* | *0.002* | *0.001* | *<0.001* | *0.002* | *0.048* |
| pH |  | 1.000 | 0.124 | -0.771** | -0.564** | -0.667** | -0.604** | -0.722** | -0.437** | -0.382** |
|  |  |  | *0.230* | *<0.001* | *<0.001* | *<0.001* | *<0.001* | *<0.001* | *<0.001* | *<0.001* |
| TBAR |  |  | 1.000 | -0.011 | -0.109 | -0.117 | -0.053 | 0.004 | 0.073 | 0.037 |
|  |  |  |  | *0.913* | *0.292* | *0.257* | *0.608* | *0.969* | *0.478* | *0.722* |
| DH% |  |  |  | 1.000 | 0.329** | 0.312** | 0.396** | 0.490** | 0.411** | 0.404** |
|  |  |  |  |  | *0.001* | *0.002* | *<0.001* | *<0.001* | *<0.001* | *<0.001* |
| ABTS |  |  |  |  | 1.000 | 0.478** | 0.463** | 0.498** | 0.188 | 0.347** |
|  |  |  |  |  |  | *<0.001* | *<0.001* | *<0.001* | *0.067* | *<0.001* |
| DPPH |  |  |  |  |  | 1.000 | 0.377** | 0.564** | 0.356** | 0.267** |
|  |  |  |  |  |  |  | *<0.001* | *<0.001* | *<0.001* | *0.008* |
| Glucosidase |  |  |  |  |  |  | 1.000 | 0.521** | 0.381** | 0.324** |
|  |  |  |  |  |  |  |  | *<0.001* | *<0.001* | *0.001* |
| Amylase |  |  |  |  |  |  |  | 1.000 | 0.336** | 0.306** |
|  |  |  |  |  |  |  |  |  | *0.001* | *0.002* |
| Caco2 |  |  |  |  |  |  |  |  | 1.000 | 0.432** |
|  |  |  |  |  |  |  |  |  |  | *<0.001* |
| MCF7 |  |  |  |  |  |  |  |  |  | 1.000 |
|  |  |  |  |  |  |  |  |  |  |  |

** Correlation is significant at the 0.01 level.

* Correlation is significant at the 0.05 level.

Pearson’s test in fermented fish sausage only
